# The effect of primary tumor radiotherapy in patients with Unresectable stage IV Rectal or Rectosigmoid Cancer: a propensity score matching analysis for survival

**DOI:** 10.1186/s13014-020-01574-8

**Published:** 2020-05-27

**Authors:** Gang Wang, Wenling Wang, Haijie Jin, Hongmin Dong, Weiwei Chen, Xiaokai Li, Guodong Li, Leilei Li

**Affiliations:** Department of Abdominal Oncology, The Affiliated Hospital of Guizhou Medical University, Guizhou Cancer Hospital, Guiyang, 550004 People’s Republic of China

**Keywords:** Stage IV rectal cancer, Primary tumor radiotherapy, Propensity score matching

## Abstract

**Background:**

To evaluate the impact of primary tumor radiotherapy on survival in patients with unresectable metastatic rectal or rectosigmoid cancer.

**Methods:**

From September 2008 to September 2017, 350 patients with unresectable metastatic rectal or rectosigmoid cancer were retrospectively reviewed in our center. All patients received at least 4 cycles of chemotherapy and were divided into two groups according to whether they received primary tumor radiotherapy. A total of 163 patients received primary tumor radiotherapy, and the median radiation dose was 56.69 Gy (50.4–60). Survival curves were estimated with the Kaplan–Meier method to roughly compare survival between the two groups. Subsequently, the 18-month survival rate was used as the outcome variable for this study. This study mainly evaluated the impact of primary tumor radiotherapy on the survival of these patients through a series of multivariate Cox regression analyses after propensity score matching (PSM).

**Results:**

The median follow-up time was 21 months. All 350 patients received a median of 7 cycles of chemotherapy (range 4–12), and 163 (46.67%) patients received primary tumor radiotherapy for local symptoms. The Kaplan–Meier survival curves showed that the primary tumor radiotherapy group had a significant overall survival (OS) advantage compared to the group without radiotherapy (20.07 vs 17.33 months; *P* = 0.002). In this study, the multivariate Cox regression analysis after adjusting for covariates, multivariate Cox regression analysis after PSM, inverse probability of treatment weighting (IPTW) analysis and propensity score (PS)-adjusted model analysis consistently showed that primary tumor radiotherapy could effectively reduce the risk of death for these patients at 18 months (HR: 0.62, 95% CI 0.40–0.98; HR: 0.79, 95% CI: 0.93–1.45; HR: 0.70, 95% CI 0.55–0.99 and HR: 0.74, 95% CI: 0.59–0.94).

**Conclusion:**

Compared with patients with stage IV rectal or rectosigmoid cancer who did not receive primary tumor radiotherapy, those who received primary tumor radiotherapy had a lower risk of death. The prescription dose (59.4 Gy/33 fractions or 60 Gy/30 fractions) of radiation for primary tumors might be considered not only to relieve symptoms improve the survival of patients with inoperable metastatic rectal or rectosigmoid cancer.

## Background

From the cancer statistical data of 2019, the incidence rate of colorectal cancer was 39.42 per 100,000 population in the U.S. [1], and the rate was 27.47 per 100,000 people in China [2]. The proportion of rectal cancer among colorectal cancers was 49.7% in China, which is higher than the corresponding 30.4% in the U.S. Approximately 25% of colorectal cancer patients present have overt metastases, and an additional 25–35% of patients will develop metastases during the course of their disease [3]. Approximately 80–90% of patients with metastatic colorectal cancer were not able to undergo a radical surgery of metastatic lesions [3, 4], therefore a surgical removal to the primary tumor was considered unnecessary, which leads to the fact that most of stage IV rectal cancer are always with primary lesion in their whole survival time. Benefitting from the combination therapies of chemotherapy and targeted drugs, unresectable stage IV colorectal cancer normally has a median survival time of 20.7–33.4 months, which has been reported by several classical studies [5–8]. At present, systemic chemotherapy is still the preferred treatment for stage IV unresectable colorectal cancer. Radiotherapy or resection of the primary tumor is only recommended for patients with primary tumor progression by the National Comprehensive Cancer Network (NCCN) guidelines [9]. These patients usually have typical local symptoms such as obstruction, bleeding, and pain. Although there is some controversy, most of the studies show that resection of the primary tumor without metastasectomy not only relieves pelvic symptoms in patients but also improves their survival [10–12]. However, few studies have explored whether radiotherapy for primary tumors can also improve the survival of these patients. To provide more meaningful clinical evidence to answer this question, in this study, we retrospectively analyzed 350 patients with stage IV unresectable rectal or rectosigmoid cancer using propensity score matching (PSM) analyses to explore whether there were any survival benefits in patients who received primary tumor radiotherapy.

## Methods

**Fig. 1:**
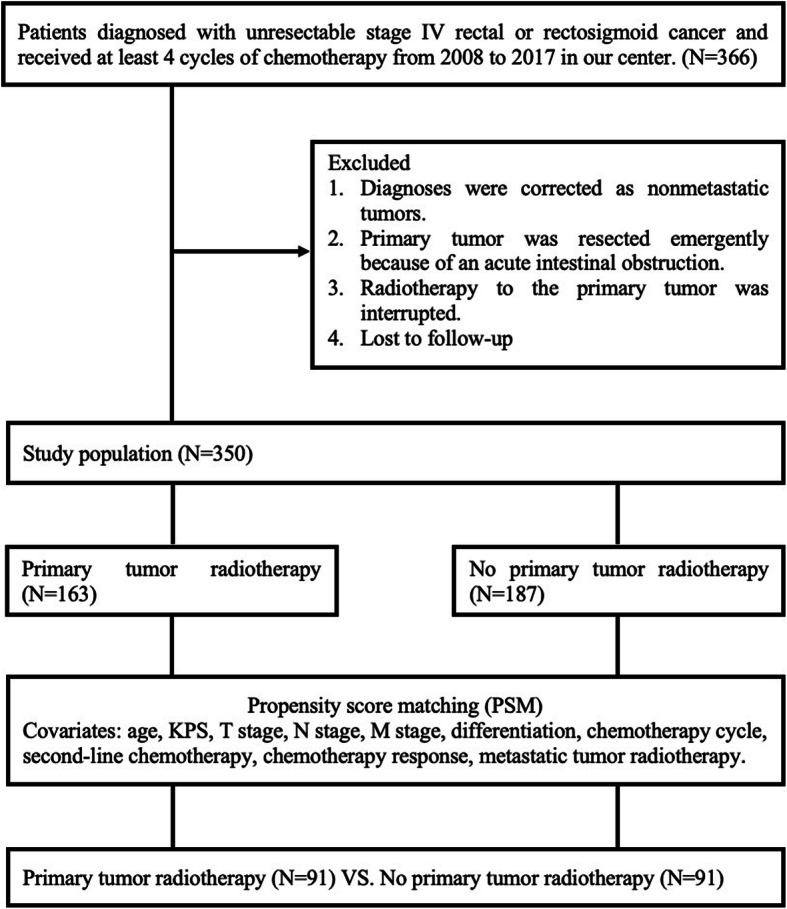
Flow diagram of the PSM process

We retrospectively reviewed 366 patients who were initially diagnosed with stage IV unresectable rectal or rectosigmoid cancer from September 2008 to September 2017 in our center. All patients received at least 4 cycles of chemotherapy, some of whom had significant local pelvic symptoms and received primary tumor radiotherapy. Four patients’ diagnoses were corrected as nonmetastatic patients by review, 3 patients underwent emergency resection of the primary tumor because of an acute intestinal obstruction, 4 patients discontinued primary tumor radiotherapy, and 5 patients were lost to follow-up. The final analysis included 350 patients, and the details of the PSM process are demonstrated in Fig. 1. Of these 350 patients, 254 were male, and 96 were female; 266 were diagnosed with rectal cancer, and 84 had rectosigmoid cancer. The numbers of patients with stage IVa, IVb and IVc disease were 180, 146 and 24, respectively, according to the 8th edition of the American Joint Commission on Cancer (AJCC) Cancer Staging Manual. The whole group of patients received chemotherapy (FOLFOX4/FOLFOX6), and the average number of chemotherapy cycles was 7 cycles. Seventy-four patients received second-line chemotherapy after disease progression. The response to chemotherapy was assessed by the Response Evaluation Criteria in Solid Tumors (RECIST); partial response (PR) or stable disease (SD) was defined as a good response to chemotherapy, and progressive disease (PD) was defined as a poor response. Because of typical local pelvic symptoms such as pain, bleeding, and incomplete obstruction, 163 patients received primary tumor radiotherapy while receiving chemotherapy. Intensity-modulated radiotherapy (IMRT) was used as primary tumor radiotherapy. Primary tumors included intestinal tumors and metastatic lymph nodes confirmed by pelvic computed tomography (CT) or magnetic resonance imaging (MRI). Radiotherapy was administered at doses of 1.8 or 2.0 Gy/day and delivered 5 days per week for a total dose of 59.4 or 60 Gy. The pelvic lymph drainage area (presacral space, internal iliac, obturator, mesorectum) within 2 cm above and below the primary tumor received 45–50.4 Gy. The patient and treatment characteristics are shown in Table 1.

**Table 1:** Comparison of clinical and treatment characteristics between the patients with primary tumor radiation and those without

Variable	Primary tumor radiotherapy				***P***-value
	No (187)		Yes (163)		
	**Number**	**%**	**Number**	**%**	
**Age**					0.460
< 60 years	90	48.13	72	44.17	
≥ 60 years	97	51.87	91	55.83	
**Sex**					0.879
Male	136	72.73	118	72.39	
Female	51	27.27	45	27.61	
**KPS**^a^					0.037
70–80	120	64.17	122	74.85	
90–100	67	35.83	41	25.15	
**Primary Site**					0.056
Rectum	134	71.66	132	80.98	
Rectosigmoid	53	28.34	31	19.02	
**T Stage**					0.043
T2	10	5.35	14	8.59	
T3	86	45.99	90	55.21	
T4	91	48.66	59	36.20	
**N Stage**					< 0.001
N0	10	5.35	40	24.54	
N1	43	22.99	71	43.56	
N2	44	23.53	42	25.77	
N+	77	41.18	9	5.52	
Nx	13	6.95	1	0.61	
**M Stage**					0.880
M1a	98	52.41	82	50.31	
M1b	77	41.17	69	42.33	
M1c	12	6.42	12	7.36	
**Differentiation**					0.013
Well	20	10.70	8	4.91	
Moderate	94	50.27	104	63.80	
Poor	61	32.62	49	30.06	
Unknown	12	6.42	2	1.23	
**Chemotherapy Cycle**					0.019
4–8 cycles	142	75.94	105	64.42	
9–12 cycles	45	24.06	58	35.58	
**Second-line Chemotherapy**					0.014
No	156	83.42	120	73.62	
Yes	31	16.58	43	26.38	
**Chemotherapy Response**					0.279
Poor	83	44.39	63	38.65	
Good	104	55.61	100	61.35	
**Metastatic Tumor Radiotherapy**					0.201
Yes	90	48.13	90	55.21	
No	97	51.87	73	44.79	

^a^*KPS* Karnofsky Performance Status

The follow-up period was defined as the time from the confirmed diagnosis of metastatic colorectal cancer until death or at least 18 months after the confirmed diagnosis. The survival time of the two groups was compared with the Kaplan-Meier method. The outcome variable was the 18-month survival rate, and whether the primary tumor was treated with radiation was used as the exposure variable. Covariates that may be related to outcome variables were screened for by referring to previous literature, clinical experience, and univariate Cox regression analysis. Multivariate Cox regression analysis was used to identify the independent effects of exposure variables on the outcome variable after adjusting for relevant covariates. This study was a retrospective observational study, not a randomized controlled trial (RCT), so selection bias was inevitable. To minimize the effect of bias, the propensity score matching (PSM) method can achieve a similar randomization effect, further verifying the previous analysis results. The matching algorithm used binary logistic regression, and the caliper value was set to 0.05. Given that PSM can cause sample loss, this study used inverse probability of treatment weighting (IPTW) as a sensitivity analysis to assess the stability of the results. Moreover, we further verified the results by adjusting the propensity score analysis (PS-adjusted model). Statistical analyses were performed using SPSS software (version 24.0, SPSS, Chicago, IL, USA) and R software.

## Results

The median follow-up time was 21 months. Patients who received primary tumor radiotherapy had more cycles of chemotherapy (35.58% vs 24.06%; *P* = 0.019) and were more likely to receive second-line chemotherapy (26.38% vs 16.58%; *P* = 0.014) than those who did not receive primary tumor radiotherapy. Patients with a lower Karnofsky performance status (KPS) score, moderate differentiation and T3 stage constituted a higher percentage in the primary tumor radiotherapy group. All of the other characteristics were similar between groups (Table 1).

**Fig. 2:**
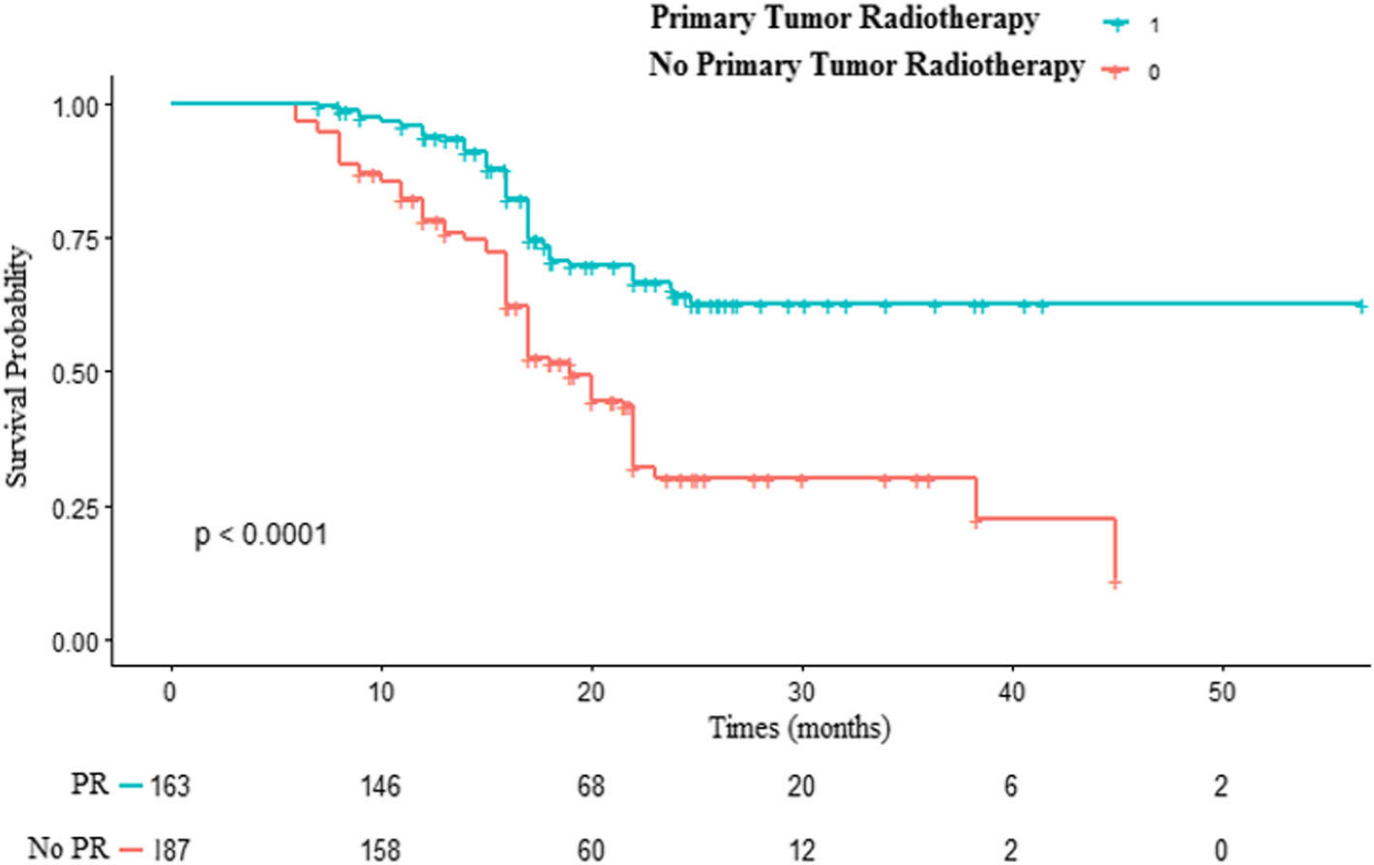
Kaplan-Meier curves for primary tumor radiotherapy and no primary tumor radiotherapy

The Kaplan–Meier survival curves showed that the primary tumor radiotherapy group had a significant overall survival (OS) advantage compared to the group without radiotherapy (20.07 vs 17.33 months; *P* = 0.002; Fig. 2). The 18-month survival rates were 73.01 and 42.25%, respectively, for the groups with and without primary tumor radiotherapy.

**Fig. 3:**
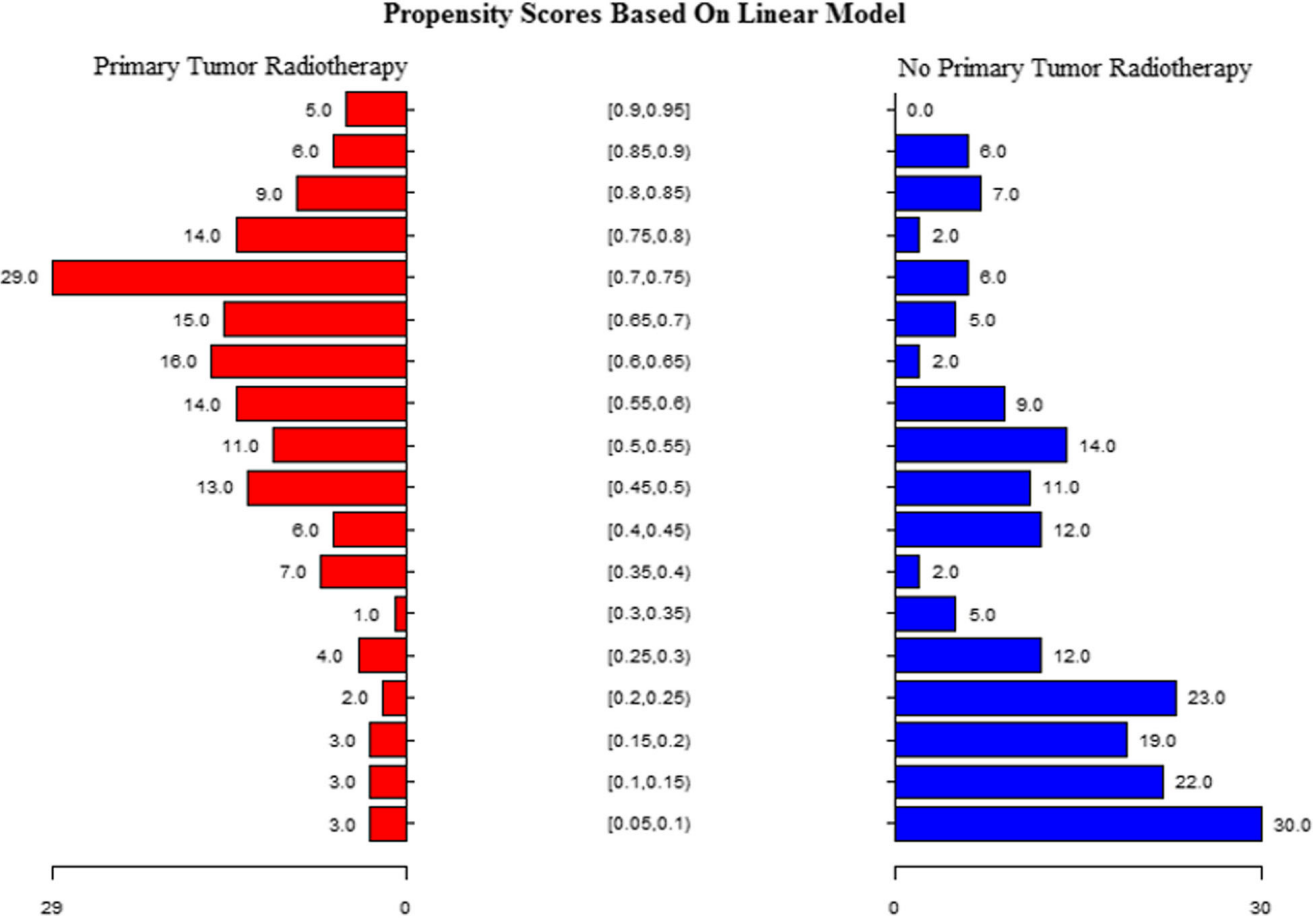
Propensity scores based on the linear model

Univariable Cox regression analysis was performed to assess the associations between covariates and the 18-month survival rate, and the results are displayed in Table 2. The possible protective factors based on the univariable Cox regression analysis include older age, more chemotherapy cycles, second-line chemotherapy, better chemotherapy response, metastatic lesions treated with radiotherapy, and primary tumor treated with radiotherapy. The possible risk factors include a higher T stage, N stage, and M stage. Based on the univariate Cox regression analysis and the distribution of related factors in the two groups of patients, 10 variables (including age, KPS, T stage, N stage, M stage, differentiation, chemotherapy cycle, second-line chemotherapy, chemotherapy response, radiotherapy for metastatic tumor) were selected as the covariates that needed to be adjusted for in subsequent multivariate Cox regression analysis. After adjusting for the above covariates in the multivariate Cox regression analysis, the primary tumor radiotherapy group had a lower risk of death than the group without primary tumor radiotherapy (HR: 0.62, 95% CI 0.40–0.98; Table 4). The 10 variables were included in propensity score matching. There were 91 matched patients in each group after 1:1 individual matching without replacement. The matching situation is shown in Fig. 3, and the clinicopathological features are presented in Table 3. The primary tumor radiotherapy group still showed a lower risk of death than the group without primary tumor radiotherapy after propensity score matching (HR: 0.79, 95% CI: 0.93–1.45; Table 4). The sensitivity analyses using propensity score–based IPTW and PS-adjusted models yielded similar results (Table 4). The results from our sensitivity analyses were consistent with our primary analysis findings. Patients treated with primary tumor radiotherapy in this study had a lower risk of death than those treated without radiotherapy (HR: 0.70, 95% CI 0.55–0.99 and HR: 0.74, 95% CI: 0.59–0.94).

**Table 2:** Univariable Cox regression analysis of factors affecting survival at 18 months in the whole group of patients

Factors	Number (%)	HR (95% CI)	***P*** value
**Age**			
< 60 years	162 (46.29)	Reference	
≥ 60 years	188 (53.71)	0.98 (0.97, 1.00)	0.0297
**Sex**			
Male	254 (72.57)	Reference	
Female	96 (27.43)	0.88 (0.62, 1.26)	0.4927
**KPS**			
70–80	242 (69.14)	Reference	
90–100	108 (30.86)	1.00 (0.98, 1.03)	0.7220
**Primary Site**			
Rectum	266 (76.00)	Reference	
Rectosigmoid	84 (24.00)	1.12(0.53,1.18)	0.6210
**T Stage**			
T2	24 (6.86)	Reference	
T3	176 (50.29)	2.86 (1.05, 7.83)	0.0406
T4	150 (42.86)	3.16 (1.15, 8.64)	0.0253
**N Stage**			
N0	50 (14.29)	Reference	
N1	114 (32.57)	0.88 (0.44, 1.75)	0.7192
N2	86 (24.57)	2.03 (1.05, 3.93)	0.0350
N+	86 (24.57)	4.09 (2.21, 7.59)	< 0.0001
Nx	14 (4.00)	7.01 (3.14, 15.66)	< 0.0001
**M Stage**			
M1a	180 (51.43)	Reference	
M1b	146 (41.71)	1.09 (0.77, 1.54)	0.6163
M1c	24 (6.86)	2.39 (1.46, 3.91)	0.0006
**Differentiation**			
Well	28 (8.00)	Reference	
Moderate	198 (56.57)	0.28 (0.17, 0.47)	< 0.0001
Poor	110 (31.43)	1.00 (0.61, 1.65)	0.9877
Unknown	14 (4.00)	0.95 (0.22, 4.07)	0.9444
**Chemotherapy Cycles**			
4–8	247(70.57)	Reference	
9–12	103(29.43)	0.79 (0.69, 0.90)	0.0002
**Second-line Chemotherapy**			
No	276 (78.86)	Reference	
Yes	74 (21.14)	0.37 (0.22, 0.63)	0.0002
**Chemotherapy Response**			
Poor	146 (41.71)	Reference	
Good	204 (58.29)	0.82 (0.59, 1.13)	0.0217
**Metastatic Tumor radiotherapy**			
Yes	180 (51.43)	Reference	
No	170 (48.57)	1.53 (1.11, 2.11)	0.0102
**Primary Tumor Radiotherapy**			
No	187 (53.43)	Reference	
Yes	163 (46.57)	0.39 (0.28, 0.56)	< 0.0001

**Table 3:** Clinicopathological features between the two groups after propensity score matching

Variable	Primary Tumor Radiotherapy				Standardized difference	***P*** value
	No (91)		Yes (91)			
	Number	%	Number	%		
**Age**					0.0224	1.0000
< 60 years	43	47.3	44	48.4		
≥ 60 years	48	52.7	47	51.6		
**KPS**						0.1700
70	14	15.4	32	35.2	0.4674	
80	65	71.4	42	46.2	0.5313	
90	12	13.2	14	15.4	0.0628	
100	0	0.0	3	3.3	0.2611	
**T Stage**						0.1156
T2	10	11.0	5	5.5	0.2008	
T3	43	47.3	56	61.5	0.2898	
T4	38	41.8	30	33	0.1825	
**N Stage**						0.1250
N0	10	11.0	15	16.5	0.1601	
N1	44	48.4	29	31.9	0.3412	
N2	25	27.5	38	41.8	0.3037	
N+	9	9.9	7	7.7	0.0777	
Nx	3	3.3	2	2.2	0.0673	
**M Stage**						0.9395
M1a	50	54.9	50	54.9	0.0000	
M1b	37	40.7	36	39.6	0.0224	
M1c	4	4.4	5	5.5	0.0507	
**Differentiation**						0.3212
Well	7	7.7	6	6.6	0.0427	
Moderate	53	58.2	52	57.1	0.0222	
Poor	28	30.8	33	36.3	0.1166	
Unknown	3	3.3	0	0	0.2611	
**Chemotherapy Cycles**						0.0940
4	20	22.0	25	27.5	0.1276	
5	5	3.3	0	0	0.2611	
6	22	24.2	19	20.9	0.0790	
8	20	22.0	14	15.4	0.1698	
10	5	5.5	20	22	0.4932	
12	19	20.9	13	14.3	0.1739	
**Second-line Chemotherapy**					0.0000	1.0000
No	74	81.3	74	81.3		
Yes	17	18.7	17	18.7		
**Chemotherapy Response**					0.1562	0.3680
Poor	42	46.2	35	38.5		
Good	49	53.8	56	61.5		
**Metastatic Tumor Radiotherapy**					0.0880	0.6565
Yes	44	48.4	48	52.7		
No	47	51.6	43	47.3

**Table 4:** Various analysis models for the risk of death at 18 months in the two groups of patients

Methods	Primary Tumor Radiotherapy		
	No	Yes	
		HR (95% CI)	***P*** value
COX adjusted^a^	Reference	0.62(0.40–0.98)	0.0394
PSM model	Reference	0.79(0.93–1.45)	NS
IPTW model	Reference	0.70(0.55–0.99)	0.0436
PS-Adjusted^b^	Reference	0.74(0.50–0.94)	0.0254

^a^Adjusted covariates included age, KPS, T stage, N stage, M stage, differentiation, chemotherapy cycle, second-line chemotherapy, chemotherapy response, and metastatic lesion radiotherapy

^b^Propensity scores were adjusted

*NS* Not significant

## Discussion

With the application of oxaliplatin and irinotecan in combination with the fluorouracil regimen, the survival time of stage IV colorectal cancer ranged from 16 to 20 months [13–15]. After entering the era of targeted drugs combined with chemotherapy, the survival time of stage IV colorectal cancer has been significantly improved to 20.7–33.4 months.

For patients with stage IV colorectal cancer who cannot be cured by radical surgery, in general, resection or radiotherapy was used as a local treatment to relieve local obstruction, hemorrhage and pain. More clinical studies focus on the benefits of primary tumor resection alone. Although there are still controversies at present [16, 17], most of the existing clinical studies show that resection of the primary tumor alone can not only reduce the incidence of local complications [18] but also seems to be beneficial in terms of patient survival [11, 19, 20]. However, there are limited data regarding the effect of primary tumor radiotherapy in stage IV unresectable rectal or rectosigmoid cancer, and most of these studies mainly observed the palliative effect [21–24]. To the best of our knowledge, very few studies have explored the effects of primary tumor radiotherapy on the survival of metastatic rectal cancer. For clinical researchers, the main reason is that there are many factors that can affect the survival of patients with stage IV rectal cancer, and there are large individual differences. In retrospective observational studies, conventional multivariate regression analysis has difficulty effectively removing interference of confounding factors and selection bias from the results, which makes the analysis results lack reliability and consistency. Moreover, it is very difficult to implement such randomized controlled trials; for example, two previous trials (NCT01086618 and NCT01978249) were terminated due to recruitment problems. This study designed a series of analyses based on PSM to minimize interference from other confounding factors and selection bias on the research results.

In previous clinical studies on primary tumor radiotherapy for metastatic rectal cancer, the radiotherapy doses were generally low. Sager et al. reviewed many studies in which the radiotherapy dose delivered to the primary tumors ranged from 25 to 50 Gy [25]. When the α/β of the tumor was assumed to be 10 Gy for the biologically equivalent dose (BED), the BED of the above studies ranged from 37.5 to 53.1 Gy. In this study, the radiation dose of the primary tumor was significantly higher than that in previous clinical studies. Overall, 78% of patients completed the prescription dose (59.4 Gy in 33 fractions or 60 Gy in 30 fractions) of radiotherapy, the average radiation dose was 56.69 Gy, and the average BED was 67 Gy. Previous studies showed that a prescription dose of 54 Gy to 60 Gy (BED = 65 to 72 Gy) delivered to rectal tumors would achieve a significant tumor regression effect, and the percentage of patients with tumor regression grade (TRG) 1 and 2 was approximately 60 to 63.9% [26, 27].

In this study, Kaplan-Meier survival analysis showed that the median survival times of the primary tumor groups with and without radiotherapy were 20.07 ± 8.98 months and 17.33 ± 7.34 months, respectively. This was consistent with previous studies on stage IV colorectal cancer patients who only received chemotherapy (median survival was 16 to 20 months), so we decided to use the 18-month survival rate as the outcome variable in this study. Furthermore, in this study, the priori selection of covariates was based on previous studies and the experience of the authors but also considered the results of the univariate analysis. Subsequently, multivariate Cox regression analysis after adjusting for covariates, analysis after PSM, IPTW analysis and PS-adjusted model analysis were performed to examine the reliability of the results. All analyses consistently showed that primary tumor radiotherapy could effectively reduce the risk of death for these patients at 18 months. According to the results of the different analysis models above, although the hazard ratio (HR) increased significantly, the reduction in the risk of death did not change, and the range of the confidence interval gradually narrowed. Our results became more conservative and accurate with the IPTW and PS-adjusted model analyses. A retrospective observational study similar to this study showed that palliative radiotherapy could improve the survival of patients with metastatic rectal cancer [28]. However, several deficiencies exist in the study. The study did not further analyze the location of the lesion (primary or metastatic) targeted by palliative radiotherapy, and it did not consider the dose. Chemotherapy, as an important factor affecting the survival of patients with metastatic rectal cancer, was not analyzed in this study. These deficiencies have been corrected in this study.

There are still some shortcomings and limitations in this study: (1) the time range of eligible patients included in this retrospective study was from September 2008 to September 2017. During this period, the price of bevacizumab and cetuximab in China were high, and these drugs were not covered by local health care insurance. Patients could rarely afford these medications, so this study did not select patients who received bevacizumab or cetuximab. The lack of targeted drugs will definitely reduce the survival benefit of patients and may affect the benefits of radiotherapy for primary tumors. (2) Compared with the 12 cycles recommended by the guidelines, the median number of chemotherapy cycles in this study was relatively low, at only 7 cycles. Fewer chemotherapy cycles will reduce the therapeutic efficacy for all patients and may have an uncertain impact on the benefits of primary tumor radiotherapy. (3) This study was a real-world study (observational clinical study). There might be some confounding factors outside of clinical cognition and previous literature reports that may affect the accuracy of the research results.

## Conclusions

In this study, compared with patients with stage IV rectal or rectosigmoid cancer who did not receive primary tumor radiotherapy, those who received primary tumor radiotherapy had a reduced risk of death for 18 months. The dose pattern of 59.4 Gy in 33 fractions or 60 Gy in 30 fractions was acceptable during concurrent chemotherapy. These doses of radiation for primary tumors might be considered not only to relieve symptoms but also to improve the survival of patients with inoperable metastatic rectal or rectosigmoid cancer.

## Data Availability

The datasets generated and/or analyzed during the current study are not publicly available since the participants did not consent to share the data with third parties.

## Abbreviations

AJCC: American joint commission on cancer
BED: Biologically equivalent dose
CT: Computed tomography
Gy: Gray
HR: Hazard ratio
IMRT: Intensity-modulated radiotherapy
IPTW: Inverse probability of treatment weighting
KPS: Karnofsky performance status
MRI: Magnetic resonance imaging
NCCN: National comprehensive cancer network
OS: Overall survival
PD: Progressive disease
PR: Partial response
PS: Propensity score
PSM: Propensity score matching
RCT: Randomized controlled trial
RECIST: Response evaluation criteria in solid tumors
SD: Stable disease
TRG: Tumor regression grade
